# Complement in the Initiation and Evolution of Rheumatoid Arthritis

**DOI:** 10.3389/fimmu.2018.01057

**Published:** 2018-05-28

**Authors:** V. Michael Holers, Nirmal K. Banda

**Affiliations:** Division of Rheumatology, Department of Medicine, University of Colorado Anschutz Medical Campus, Aurora, CO, United States

**Keywords:** complement, arthritis, classical pathway, lectin pathway, alternative pathway, mannose-binding protein-associated serine proteases, inflammation

## Abstract

The complement system is a major component of the immune system and plays a central role in many protective immune processes, including circulating immune complex processing and clearance, recognition of foreign antigens, modulation of humoral and cellular immunity, removal of apoptotic and dead cells, and engagement of injury resolving and tissue regeneration processes. In stark contrast to these beneficial roles, however, inadequately controlled complement activation underlies the pathogenesis of human inflammatory and autoimmune diseases, including rheumatoid arthritis (RA) where the cartilage, bone, and synovium are targeted. Recent studies of this disease have demonstrated that the autoimmune response evolves over time in an asymptomatic preclinical phase that is associated with mucosal inflammation. Notably, experimental models of this disease have demonstrated that each of the three major complement activation pathways plays an important role in recognition of injured joint tissue, although the lectin and amplification pathways exhibit particularly impactful roles in the initiation and amplification of damage. Herein, we review the complement system and focus on its multi-factorial role in human patients with RA and experimental murine models. This understanding will be important to the successful integration of the emerging complement therapeutics pipeline into clinical care for patients with RA.

## Complement System and its Activation

It was Buchner who, at the University of Munich, discovered a blood born substance that was able to destroy bacteria. He named it “alexin.” The term “complement” was subsequently introduced by Ehrlich as part of his grand model of the immune system (1–6). Although initially considered primarily in the context of resistance to infection, the complement system, as an important arm of the innate immune system, has been long recognized to play an important role in tissue damage in many autoimmune diseases, including rheumatoid arthritis (RA). Thus, the complement system responds not only to microorganisms but also mediates inflammation through the orderly activation of a cascade of multi-protein enzymes and proteases.

Key functions of the complement system include clearance of foreign microorganisms through specific recognition, opsonization, and lysis (7). The system also plays major roles in the clearance of circulating immune complexes (CICs), apoptotic cells, apoptotic bodies, and dead cells (8, 9). Out of three different types of CICs (small, intermediate, and large), intermediate CICs typically cause the most damage as they get trapped in the tissues or in the joints. These protective functions provide potent properties for the benefit of the host, even in the absence of an adaptive immune response.

Although the complement system limits its pro-inflammatory and anti-inflammatory activities, through action of many inhibitors under normal physiological conditions, these natural complement inhibitors are not enough when the complement system gets over-activated during acute inflammatory conditions and thereby causes more damage than good. The functions of the complement system are not only limited to serum or plasma where these are found in abundance but to each and every tissue or organ of the body which are the direct target of various complement components.

**Figure 1:**
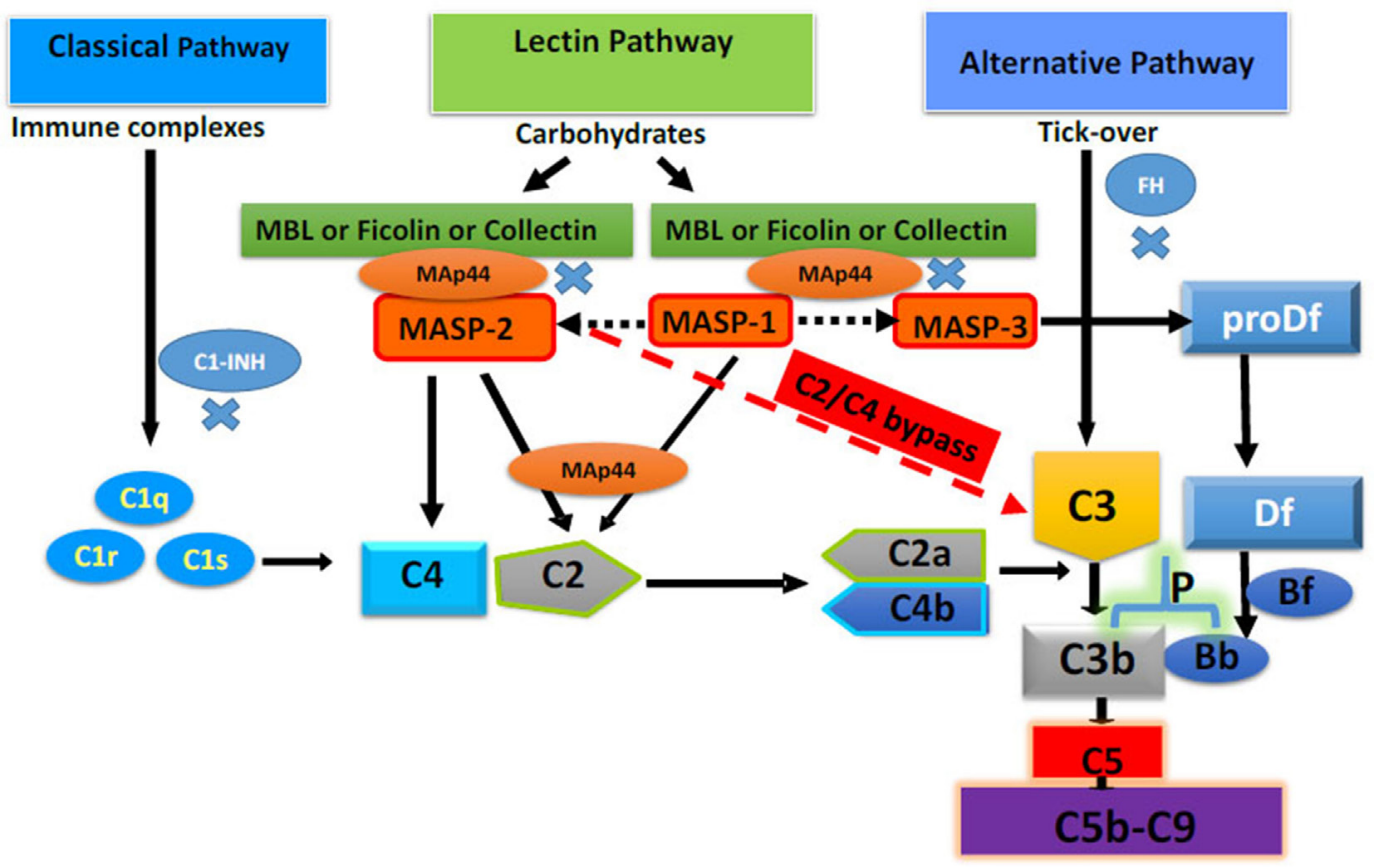
Complement system with four different activating pathways, i.e., classical, lectin, alternative, and C2/C4 bypass. Only major complement inhibitors of the classical pathway, lectin pathway, and alternative pathway, i.e., Cl-INH, mannose-binding lectin-associated protein of 44 kDa a.k.a. MBL/ficolin/CL-11-associated protein-1, and FH, respectively, have been shown. All pathways converse to cleave C3 and C5 to initiate the terminal pathway of the complement system, i.e., membrane attack complex (C5b-9). Adapted from Ref. (10). Copyright 2017. The American Association of Immunologists, Inc.

Most proteins of the complement system are normally present in the circulation in an inactive (zymogen) form to be activated *via* proteolytic processing upon the recognition of danger. Interestingly, there exists multiple pathways by which the complement system may be activated, each employing different recognition molecules, which underscores its great complexity. The complement system is activated by three different major pathways: the classical pathway (CP), the lectin pathway (LP), and the alternative pathway (AP) and one minor pathway, the C2/C4 bypass (10) (Figure 1). All of these pathways are activated by various antibodies, ICs, molecules or microorganisms, or spontaneously as discussed below.

### Classical Pathway Activation

The CP is activated by binding of C1q to the heavy-chain crystallizable fragment (Fc) domain of immunoglobulin (Ig). In mice, IgM, IgG1, IgG2a, and IgG2b all have complement activation sites, and these can form CICs when combined with an antigen and complement. C1q leads to the activation of C1r, followed by activation of C1s. C1s cleaves and activates C4 into C4a and C4b and also C2 into C2a and C2b, leading to the formation of C4b2a (CP C3 convertase), which itself cleaves C3 into C3a and C3b (11). C3b further binds to C4b2a to generate the C5 convertase of the CP. This initiates the formation of C5b-9, the membrane attack complex (MAC) (12).

Through its recognition mechanisms, C1 can help to distinguish self from non-self, which is important for the maintenance of self-tolerance and homeostasis (13). Conversely, its pathologic activation has been implicated in many inflammatory and autoimmune diseases, and its activation is limited by C1 esterase inhibitor (C1-INH) (14). Recently, it has been shown that C4a is a ligand for protease-activated receptor (PAR) 1 and PAR4, extending the direct link between the complement and coagulation systems (15). In addition, MAC assembly has been shown on the surface of parasites, and to eliminate Gram-negative bacteria and unwanted host cells (16–18). The MAC can rupture cells with varied composition of lipids and once MAC assembly initiates on cell surfaces other factors can still block it (16). Interestingly, sub-lytic levels of MAC either causes the release of pro-inflammatory mediators or in other circumstances acts to increases the protection of cells to avoid further innocent bystander cell lysis (19, 20).

### Lectin Pathway Activation

The recognition components of the LP, mannose-binding lectins (MBLs), ficolins (FCNs), and collectins (CLs) bind directly to microbial and other surfaces with exposed carbohydrates and *N*-acetyl groups and activate complement *via* MBL-associated serine proteases (MASPs) (21–23).

Ficolins, which contain a carbohydrate recognition domain (CRD), consist of collagen-like and fibrinogen-like domains and preferentially bind to *N*-acetylglucosamine (GlcNAc) (24–26). There are two mouse FCNs: ficolin A (FCN A) (27) and ficolin B (FCN B) (28); by contrast, humans express three FCNs; ficolin M (FCN-M a.k.a. FCN 1), ficolin L (FCN-L a.k.a. FCN 2), and ficolin H (FCN-H a.k.a. FCN 3) (29–33). Mouse FCN A, but not FCN B, exhibits a splice variant known as FCN A variant (34). FCN A is present in the serum and expressed in liver hepatocytes (35). Mouse FCN B was originally found in the lysosomes of macrophages, similar to human FCN-M, which is also found in the secretary granules of monocytes and neutrophils (36, 37). We have reported that FCN B is also present in the circulation of mice suggesting that it is secreted from macrophages (38).

Mannose-binding lectin is a C-type lectin containing a CRD as well as a collagen-like domain (21, 22). MBL binds to mannose-containing molecules as well as *N*-acetylglucosamine (21, 22). There are two types of mouse MBLs, i.e., MBL-A and MBL-C, whereas there is only one type in human (39). Four different types of MASPs called mannose-binding lectin-associated serine protease-1 (MASP-1), mannose-binding lectin-associated serine protease-2 (MASP-2), mannose-binding lectin-associated serine protease-3 (MASP-3), and sMAP (also called Map19) (small MBL-associated protein) circulate complexed with MBL, FCNs, and CL (40). Although no specific function has been assigned to sMAP by generating *sMAP^−/−^* mice, it was shown that the expression of MASP-2 was also decreased in the sera of these mice because of the MASP-2 gene disruption (41). These authors have also shown by using sera from *sMAP^−/−^* mice that sMAP plays a regulatory role in the activation of the LP but it is not clear whether sMAP plays a regulatory role before or after the LP activation. sMAP and MASP-2 compete to bind to MBL, and sMAP has the ability to downregulate the LP (41). MAp44 (also called MAP-1), an alternatively spliced product of the *MASP-1/3* gene, is a natural inhibitor of the interactions between MBLs and FCNs and serves as a major regulator of the LP (42, 43). MASP-1, MASP-2, and MASP-3 consist of an A chain (1CUB, EGF, 2CUB, 1CCP, 2CCP, and the linker region) linked by a disulfide bond to a B-chain (serine protease domain).

Both the CP and LP share C2 and C4 complement components. Similar to the CP, the LP forms the C3 and C5 convertases leading to the formation of MAC. A recent additional breakthrough has been the finding that MASP-3, which is an alternative spliced form of *MASP-1/3* gene, is a positive regulator of the AP of the complement system (44) and MASP-3 exclusively enables FD maturation (45). It has been shown that both MASP-1 and MASP-2 can activate MASP-3, but MASP-3 in resting human blood is also present in an active form (46). *In vitro*, not *in vivo* studies, have shown that MASP-1 is essential for bacterial LPS but not Zymosan-induced AP activation (47), indicating that MASP-1 can regulate a specific AP activation mechanism but not the entire AP.

The third class of LP initiators, designated CLs, are similarly C-type lectins containing CRDs (48). Three different human CLs have been identified: Collectin-10 (a.k.a. collectin liver 1, CL-L1, or CL-10), Collectin 11 (a.k.a. collectin kidney 1, CL-K1, or CL-11), and Collectin-12 (collectin placenta 1, CL-P1, or CL-12) (49–52). CL-K1 is present in various human and mouse tissues (49, 53). It has also been shown that CL-K1 acts as a soluble pattern recognition receptor for *Mycobacterium tuberculosis* (54). Additionally, it binds to l-fucose beside other potential ligands (55). In a renal injury model, CL-11 expression was rapidly upregulated and recognized the abnormal presentation of l-fucose leading to complement activation and tissue injury (56). Interestingly, both CL-11 and MASP-2 have been shown to generate C3d on injured cells (56). All of the abovementioned collectins play an important role in an innate immunity because these can bind to the LPS from various species of bacteria (57–60).

### Alternative Pathway Activation

The original properdin-dependent pathway, now called the AP of the complement system, was discovered in the 1950s (61). The AP consists of four proteins, factor B (FB), factor D (FD), Properdin (Pf or P), and C3. In contrast to the CP and LP, the AP is activated spontaneously through hydrolysis of C3, thereby generating C3(H_2_O), which can associate with FB, resulting in the cleavage of FB into Ba and Bb by FD. Therefore, the AP does not require a specific recognition molecule in order to be activated. Nevertheless, as C3 hydrolysis (a.k.a. C3 “tick over”) is always happening regardless of the presence of FB or FD (62), the system is always poised for activation. In that process, FB binds to C3(H_2_O), and can be cleaved by FD to generate C3(H_2_O)Bb (C3 convertase). The cleavage is much slower without properdin, which is a positive regulator of the AP convertase and can also independently promote the activation of the AP on certain surfaces (63). C3b bound to Bb on a surface is a potent C3 convertase and can cleave C3 to generate more C3b and C3a. The C3 convertase can also combine with another C3b molecule forming an AP C5 convertase. The latter can start the formation of the MAC after cleaving complement component C5 into C5b and C5a. In contrast to activation, complement factor H (FH) is the natural regulator of the AP, and in addition to solution phase AP blockade the binding of this molecule to one or more of the host marker recognition sites enables it to control surface activation of the AP (64). There are also several membrane-bound inhibitory proteins described below which determine the location and activity of the complement system (65). Finally, regardless of the activation route, all of these pathways generate two major potent pro-inflammatory molecules; C3a and C5a, *via* C3 and C5 convertases, respectively, which play a vital role in the pathogenesis of arthritis.

### C4/C2 Bypass Pathway Activation

A fourth pathway has also been found to be important in the generation of complement pro-inflammatory mediators and termed the “C4/C2 bypass pathway” or “C2-independent” pathway (66). Initially, it was shown that C4 and C2 complement components were not necessary to lyse cells by the CP (67–69) despite the fact that C4 and C2 are important constituents of the C3 convertase. More recently, it was shown that the components of the LP such as MBL in the absence of C2, C4, or MASP-2 induce C3 deposition (70). This could be mediated by LP ligands such as MBL or FCNs. These could activate the AP directly *via* MASP-3, which cleaves proFD into FD (10). C3 activation in the absence of C2, C4, or MASP-2 requires FB as well as a high concentration of serum (70). C3 activation on adherent anti-collagen (anti-CII) antibodies, was also reported at a high concentration using sera from mice lacking C4 by an unknown mechanism (71). This C3 activation on adherent anti-CII antibodies was fully inhibited by an anti-FB inhibitory antibody (71), confirming specific AP activation in the absence of C4. Even in human serum lacking C4, MASP-2-dependent C3 activation was reported *via* a C4 bypass route (72). So, this C4/C2 bypass pathway is operative without forming conventional CP or LP C3 convertases. It has been shown that thrombin is capable of generating the complement activation product C5a in the complete absence of C3 (73), which represents another bypass mechanism to generate C5a and C5b. Recently it has been shown that in the absence of C4, the CP cannot be activated however, LP still retains the capacity to cleave C3 into C3a and C3b. This residual C4/C2 bypass is dependent on MASP-2 (74). This study further demonstrated that MASP-2 dependent cleavage of C3 was inhibited by MASP-2-specific inhibitors. All of these studies are consistent with the presence of a backup or bypass complement pathway that works *in vivo* in the absence of C4 or C2. Whether this pathway, like CP, LP, and AP, is controlled by the well-described complement regulators is not known. Importantly, the relative importance of the C4/C2 bypass pathway in relation to arthritis models has recently been shown (10), and other studies have revealed its importance in some ischemia/reperfusion-related models (72).

### Complement Mediators of Inflammation and Their Receptors

Regardless of the activation pathway, cleavage of C3 is followed by generation of C3a, C3b, iC3b, C3d, C5a, C5b, and MAC (75, 76). Recently, it has been shown that human plasma kallikrein directly cleaves C3 into C3a and C3b and triggers an amplification loop (77). Interestingly, the cleavage site within C3 is identical to that recognized by C3 convertase and is also inhibited by FH. The cleavage of C3 and C5 by kallikrein or thrombin appears to represent a coordination between the complement system and the coagulation pathways (78). C4 cleavage leads to the generation of C4a, another anaphylatoxin but it is not clear whether it is a chemoattractant. C3a, C4a, and C5a are called anaphylatoxins because they are able to carry out pro-inflammatory activities even at a very low concentration. Thus, complement can contribute to the inflammatory injury through many mechanisms.

Previously, it has been shown that C3a is less potent than C5a while C3a desArg (a.k.a. acylation-stimulating protein) has no inflammatory activity. It has also been shown that C3a is not a chemoattractant for neutrophils but can cause migration of eosinophils (79). But this view has shifted based on new findings. C3a binds C3aR expressed on the surface of neutrophils, eosinophils, and basophils, monocytes/macrophages, and mast cells (80, 81). C3a and C5a can bind and equally activate through their receptors C3aR and C5aR, respectively, present on the surface of basophils and mast cells (82).

C4a is very weak anaphylatoxin which is formed by the cleavage of C4 into C4a and C4b. The view whether or not C4a is a classical anaphylatoxin has been recently questioned because evidence has been provided that C4a is a ligand for PAR1 and PAR4 (15). These authors have shown that C4a showed no activity toward known anaphylatoxin receptors but it acted as a non-traditional agonist for both PAR1 and PAR4.

C5a is a cleaved by-product of C5 after complement activation. C5a is rapidly converted by carboxypeptidases to less potent C5a desArg but still has biological activity, and the view regarding C5a desArg potency has also been challenged. Most of the C5a found in the circulation is in the C5a desArg form (83). The binding affinity of C5a to C5aR been reported 100-fold higher than that of C5a desArg for C5aR (84). Although C5a is considered as the triggering molecule but it has been shown that C5a desArg also acts as an important molecule triggering of local inflammation and also maintain blood surveillance and homeostatic status. This study has elegantly shown that C5a desArg induce cell activation in even higher than C5a, which was dependent on the C5aR because it was inhibited by PMX-53, a C5aR antagonist (85). C5a acts as a chemotactic factor of neutrophils and increases neutrophil adhesion to endothelium (86, 87). C5a binds to C5aR (C5aR1 or CD88) and C5L2 (C5aR2 or GPR77) present on many cells leading to chemotaxis of inflammatory cells, vascular permeability, phagocytosis, and release of pro-inflammatory cytokines and chemokines. C5a amplifies tissue injury and inflammation by triggering release of oxygen free radicals and arachidonic acid metabolities (88). C5a is an essential component of the inflammatory response to bacterial infection. *Porphyromonas gingivalis* expresses a peptidyl arginine deiminase (PAD) with a strong preference for the C-terminal arginine of C5a, disabling protein function resulting in decreased chemotaxis of human neutrophils (89). It has been shown that C5a, released at sites of inflammation, upregulates FcγRIIIa and downregulates FcγRIIb simultaneously (90, 91).

The biological and pathological role of the second C5a receptor, C5L2 is controversial. C5L2 does not bind C3a or ASP/C3adesArg. C5L2 also binds to C5a and C5a desArg. C5a desArg binds 20-fold to 30-fold with higher affinity with C5L2 than C5aR (84, 92). Although C5L2 binds to C5a with the same high affinity as C5aR but function may depend on the cell type, species, and disease context (93). While C5aR is a G protein-coupled receptor, C5L2 is not which led to the hypothesis that C5L2 functions as a decoy receptor. This view is not universally accepted, however. Evidence has been generated linking C5L2 to both anti-inflammatory and pro-inflammatory functions (93). We have found no evidence for C5L2 playing a role in RA; however, so we will leave this fascinating topic for others to review.

Membrane attack complex formation is the final step of the terminal pathway after cleavage of C5 by C5 convertases of the CP/LP or of the AP leading to the formation of the pore consisting of C5b–C9 complement proteins (94). MAC formation may lead either to necrosis or apoptosis, in part depending on the number of pores formed. The choice of necrosis vs. apoptosis is clinically relevant as necrosis is invariably pro-inflammatory, while apoptosis can lead to the resolution of an inflammatory response (95, 96). In general, eukaryotic cells require more pores than prokaryotic cells to induce death. Interestingly, low numbers of MAC, rather than leading to cell death, induce inflammatory signaling events such as the release of TNF-α and IL-1 (97).

Additionally, complement receptor 1 (CR1 or CD35), complement receptor 2 (CR2 or CD21), complement receptor 3 (CR3 or CD11b/CD18), complement receptor 4 (or CD11c/CD18), and complement inhibitors such as FH, decay-accelerating factor (DAF or CD55), membrane cofactor protein (or CD46), and protectin (CD59) have been shown to play an important role in the complement-mediated injury and also, as we will discuss below, in the pathogenesis of arthritis.

### Measurement of Complement Activation in Inflammation

Classical pathway activity is commonly measured using the CH50 test. Here, serum can be used to lyse sheep erythrocytes coated with anti-sheep antibodies, and degree of hemolysis is measured. By contrast, the AH50 is the best screening test used to measure the proper functioning of the AP. Low levels in either test indicate a deficiency of one or more components of the CP or AP of the complement system, respectively (98). Function of the LP can be measured by enzyme-linked immunosorbent assay pre-coated with mannan particles, and here C4d bound to mannan can be measured. Furthermore, the CP component C4d has been used as a measure to explore the activation of the CP. Bb levels have been used to as a measure to explore the activation of the AP. Tissue bound or soluble MAC levels have been used as measure of the activation of all pathways of the complement system thus as the most important indicator of complement activation within a microenvironment. To measure complement activation and its split products in serum or plasma, there are excellent standard protocols. Conversely, results can be obtained by using fee for services complement focused laboratories and commercially available kits (99–102).

Most of the stable complement components are measured in serum whereas activated split products are measured in plasma (EDTA-anticoagulated blood) due to the interference of the coagulation system enzymes leading to erroneous results. Furthermore, most measurements of complement activity are focused on serum or plasma with no attention paid to specific cells or tissues (e.g., synovial fluid). Since, in clinical disease, complement causes damage locally, more work must be done to assess complement activity on tissue surfaces and within sequestered regions such as synovial fluid. Measurements of CICs along with complement products such as C1q, C3b, C3d, C3dg, and MASPs levels in the serum and synovial fluid of RA patients along with rheumatoid factor (RF), anti-citrullinated protein antibodies (ACPA), and anti-carbamylated protein (anti-CarP) antibodies can provide a better picture of the local production and their role in the RA pathogenesis. Sometimes it is hard to make any conclusion from measuring C3 or C4 levels alone using serum or plasma or synovial fluid because excessive production due to inflammation masks their consumption and results are confounded by the coagulation pathway. Furthermore, endogenous complement inhibitors of the CP, LP, and AP work only under normal physiological conditions but these are ineffective under pathophysiological conditions due to the hyper-activation of complement. Therefore, their measurement provides less useful information.

## Initiation of Rheumatoid Arthritis

Rheumatoid arthritis is a chronic inflammatory systemic disease that primarily affects peripheral joints, thereby leading to synovial inflammation followed by cartilage and bone destruction. During the development of the disease, the synovium undergoes proliferation, thickens, and incorporates a large number of infiltrating immune cells to become a new tissue called pannus that causes cartilage and bone damage (103–105). Although the exact origin or initiation or development of RA is unknown, studies have shown associations in patients with active RA with infections in the temporomandibular joints (106, 107) or in the gums due to severe periodontitis (108). Dysbiosis of the microbiome in the oral or gut regions has also been strongly associated with onset of RA (109). Furthermore, interstitial lung disease (110), infections by alphaviruses (mosquito-transmitted viruses) such as Ross River virus (111), Chikungunya virus (112), and HBV (113) are also associated with a risk for the development of RA. RA also exhibits a genetic predisposition, with approximately 50% of this genetic risk contributed by certain HLA-DR alleles (114). Many other genes have been shown to contribute to RA pathogenesis (115). Additionally, environmental exposures such as air pollution, occupational exposure to silica, active smoking, wood burning, and mineral oil have been shown to acts a risk factor for initiating and/or developing RA (116, 117). The hypothesis that smoking and pollution lead to an increased risk of RA paved the way to the hypothesis that initial inflammation and production of RA-related autoantibodies (called ACPA anti-citrullinated protein/peptide antibodies) in the lungs may lead to RA (116, 118). So far, there are no studies showing the direct migration of ACPA from lungs to the peripheral joints to precipitate disease. ACPA is the most reliable and specific biological marker to diagnose RA and these antibodies are increased in RA patients sera almost 10 years prior to clinical diagnosis (116, 118–121). Hundreds of citrullinated proteins have been found in the synovial fluid of RA patients which might contribute to the RA pathology (122–125) but why only few autoantigens such as enolase, fibrinogen, and vimentin generate autoantibodies is not clear. How these citrullinated proteins present locally in the synovial fluid interact directly with various complement proteins and activate the complement system is also unknown.

It has been shown that there is a relationship between ACPA, RF, and systemic bone loss in early RA patients (101). The presence of citrullinated antigens on the surface of osteoclastic linage cells makes these cells the main targets of circulating ACPA leading to pro-osteoclastic events (126, 127). There is an argument for complement involvement in this process. Bacterial antigens, perforin, and the MAC cause calcium influx leading to cytolysis. PAD enzymes that convert peptidylarginine into peptidyl citrulline are calcium-dependent (128–131). Interestingly, perforin and the MAC have been shown to reproduce identical patterns of hypercitrullination seen in the neutrophils present in the synovial fluid of RA patients (132). A huge number of neutrophils are present in the synovial fluid of RA patients and are the major source of intracellular citrullination and PADs for extracellular citrullination (132–136). These data suggest that citrullinated proteins along with activation of the complement system might be contributing to the initiation of RA.

### Complement Activation on Articular Cartilage Surface and in Synovium in Rheumatoid Arthritis

Earlier studies have shown that autoantibodies to type II collagen present in the serum of RA patients bind to the cartilage components or to antigen present on the surface of articular cartilage (137). Articular cartilage is a hyaline cartilage and connective tissue of the joints. The main cellular component of adult articular cartilage is the chondrocyte. These cells, which make up approximately 1% of the tissue, function to organize collagen into ordered structures and secrete extracellular matrix (ECM) components. The ECM is composed of water, collagen type II, proteoglycans, non-collagenous proteins, and glycoproteins (138, 139). Complement activation due to antibody–cartilage surface interaction in RA patients have shown the abundant co-deposition of IgGs and activated complement components (140). Interestingly chondrocytes can also synthesize complement components including C1 and C1 inhibitor (141, 142).

**Figure 2:**
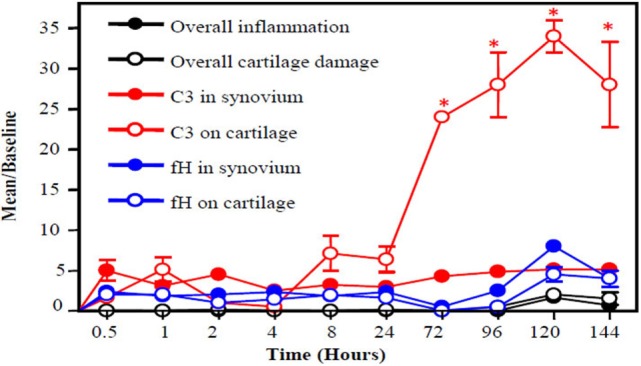
A slow snapshot of the early histopathological analysis from the knee joints of wild-type (WT) mice with collagen antibody-induced arthritis (CAIA) for C3 and FH deposition on the surface of cartilage and in the synovium. A mixture of four monoclonal antibody (mAb) to CII (8 mg/mouse) was injected i.p. to induce arthritis, and mice were sacrificed at 0.5, 1, 2, 4, 8, 24, 72, 96, 120, and 144 h later. A low level of FH was present on the surface the cartilage and in the synovium at all time points with slight non-significant increases at 72, 96, 120, and 144 h. By contrast, C3 deposition on the cartilage surface showed a large increase over baseline beginning at 8 h after injection of the anti-CII mAbs and peaking at 120 h. Thus, an imbalance exists between FH deposition and C3 deposition in the early stages of disease leading to failure to protect the knee joints in mice with CAIA. Histopathologic scoring for inflammation (black solid circle) and cartilage damage (white empty circle) from the knee joints (right and left) was performed following tissue processing and Toluidine-blue staining of sections. C3 deposition in knee joints in the synovium (red solid circle) and on the surface of cartilage (red empty circle) is illustrated, as is FH deposition in the synovium (blue solid circle) and on the surface of cartilage (blue empty circle). The data are expressed as mean of disease/baseline ± SEM (*n* = 3 each time point). Baseline = background levels of inflammation, cartilage damage, and C3 and FH deposition in the knee joints of WT mice without treatment with mAb to CII (*n* = 3). Adapted from Ref. (156). Copyright 2013. The American Association of Immunologists, Inc.

An important piece of evidence linking complement activation to pathogenesis in RA was that C1 staining was negative in normal articular cartilage and positive in degenerating cartilage biopsies from all RA patients examined (143). This study strongly implicated the involvement of the CP in the pathogenesis of RA. C3b was also present on the cartilage surface of RA patients; thus, this study clearly showed that C1s can activate the downstream complement cascade thereby causing irreversible damage. It has also been shown that the level of C1q in serum correlates with clinical disease activity (CDA) in RA patients (144–146). In mouse model of RA, C3b gets deposited first on the surface of cartilage vs. synovium and increased rapidly from 4 to 120 h (Figure 2). Interestingly, during this time, there is not enough FH availability on the surface of cartilage as well as in the synovium to protect them from complement-mediated damage (Figure 2). The presence of C2, C3, C4, and C5 in rheumatoid synovial fluid had been shown previously (147). Levels of properdin and FB of the AP were depressed. An increase in the levels of C3d, C4d, Ba, and MAC has been found in the synovial fluid of RA patients (148, 149). Normally, IgG containing ICs and also C3 split fragments can be found in the joints of more than 90% of RA patients (140, 150) and mediate complement activation. IgG with C3d has been present in the synovial fluid of RA patients and also MAC and Bb levels are elevated in the synovial fluid of RA patients (151, 152). No statistical differences in the levels of C3c and C4 in serum and in the synovial fluid of RA patients have been seen in a cross-sectional study although significant differences were seen in the CICs in both biological fluids (153). DAF expression is increased in RA synovium, while the expression of CD59 significantly decreased in the synovial lining (154, 155). The presence of split components of the complement system on the cartilage surface and in the synovium of RA patients indicate that local complement presence/and or synthesis and activation can attract macrophages for phagocytosis of chondrocytes which can further damage the cartilage and synovium.

### Complement Activation due to Glycosylation, Citrullination, and/or Carbamylation in Rheumatoid Arthritis

It has been documented that IgG is the most abundant immunoglobulin isotype comprising ~75% of the total serum immunoglobulins (157). IgG triggers its effector function (i.e., complement activation *via* its Fc). Therefore, any change due to posttranslational modification in the Fc region such as glycosylation will influence the effector function of IgG mediated by the FcγR (158–162). The glycosylation of the Fc is characterized by presence of a single chain N-linked glycan attached to each heavy chain at asparagine 297. It has been shown that the lack of fucose, sialic acid, and galactose residues on the Fc-N-linked glycans increases the inflammatory capacity of IgG in mice (163–166). IgG in RA patients contains less galactose and sialic acid (167). Interestingly, the glycosylation pattern of ACPA changes before the onset of RA skewing toward more inflammation (168, 169). Pregnancy-induced spontaneous improvement of RA as well as flares after delivery has been linked to pregnancy-related changed in the glycosylation of IgGs (170–172). This study was the first natural evidence of that changes in IgG galactosylation can cause disease pathogenicity in humans. Later on, it was shown that agalactosyl IgG is pathogenic in mice and arthritis could be transferred in mice by injecting agalactosyl IgG (173). The LP pathway component, MBL was shown to be associated with the pathogenicity of agalactosyl IgG (159). Not only the Fc but also the Fab domains of IgG has also been reported to contain N-glycosylation consensus sequences (174). It has been reported that more than 90% of ACPA-IgG molecules carry Fab glycans that are highly sialylated (175). More interestingly, ACPA-IgG purified from synovial fluid of RA patients, could even exceed 100% Fab glycosylation implying that multiple glycans can be attached to the variable domain (176). What role Fab-glycan plays in the functionality of ACPA-IgGs is unknown so far, but studies have shown that ACPA-IgG in RA have a pro-inflammatory Fc glycosylation pattern with reduced galactosylation and sialylation levels (177, 178). It has been shown that ACPA have the capacity to activate the CP and AP of the complement system (179). Thus, ACPA reduced galactosylation and sialylation have more capacity to activate the complement system to generate more vigorous effector response.

Citrullination is a normal physiological process which occurs inside apoptotic cells. Normally, apoptotic cells are scavenged by macrophages. If this system is defective, then PAD enzymes and citrullinated proteins can become externalized to influence the immune system (180).

There are five PAD isoenzymes (PAD1–4 and 6) that regulate key cellular processes (181). The PAD enzymes will citrullinate proteins by converting arginine into citrulline. Cl-amidine is the most widely used pan-PAD inhibitor (182, 183). During inflammation many cells die, and it is common to find citrullinated proteins at the inflamed sites such as in the inflamed synovium of RA patients, suggesting that ACPA could be generated as part of an immune response to self-proteins (184). In filaggrin, fibrin, and vimentin, anti-cyclic citrullinated peptide antibody (ACPA a.k.a. anti-CCP) recognizes the arginine residues modified by PAD enzymes to citrulline (185, 186). The presence of citrullinated proteins, as mentioned above, does not always mean generation of ACPA, however (180). The combination of high specificity (90–99%) and high sensitivity (66–88%) of anti-CPP for diagnosing RA, and above all, correlation with radiological damage has led to the conclusion that these antibodies have a pathological connection to the initiation of RA (185, 187–192). The clinical measure of above 20 U/ml suggests the possibility of RA. Additionally, approximately 20% RA patients are anti-CCP negative. Despite the radiological damage association with anti-CCP antibodies, the levels are not being used to determine the progression of disease since even during remission most of the subjects remains anti-CCP positive. This raises question regarding the direct pathogenicity of these antibodies in RA. It is not clear whether anti-CCP antibodies are the cause or the result of inflammation in RA patients. Whether anti-CCP antibodies are the result of defective coagulation system in RA patients is also not clear. Defective coagulation can in principle modulate the generation of anti-CCP autoantibodies. This is due to the fact that thrombin cleaves fibrinogen into fibrin followed by a clot formation but if fibrinogen is citrullinated then thrombin cannot cleave it resulting in anti-CCP immune response. The target protein is not one citrullinated protein but hundreds of citrullinated proteins as mentioned above.

One study has shown that anti-CCP antibodies activate the complement system *in vitro via* the CP and the AP but not by the LP in RA (179). In this study, anti-CCP antibodies from all 60 patients activated the complement system. This important observation leads to the evidence that complement activation can play very important role in the pathogenesis of RA in ACPA-positive patients but not all RA patients are ACPA-positive. Later on, it has been shown that citrullination locally in the joints can increase inflammation indicating the direct target of ACPA (193) and it will be consistent with the accepted paradigm that complement activation at the site of antibody recognition of citrullinated antigens can cause damage.

Furthermore, IgM RF and IgA RF amplify complement activation mediated by ACPA-IC (194). These authors concluded that ACPA-IC incorporating IgM or IgA RF participate in the triggering of the inflammation-promoting activation of complement cascades occurring in RA joints. The ACPA test has been used to classify the RA into two disease subsets, i.e., ACPA-positive (which includes the HLA-DR shared epitope subset) and ACPA-negative (no HLA shared epitope association is present) (195). These authors concluded that ACPA-positive RA is genetically different from the ACPA-negative RA. The possibility remains that ACPA-positive and ACPA-negative RA patients have differential level of IgG galactoyslation and carbamylation patterns, thereby activating different pathways of the complement system. This area has not been explored in-depth and it could provide some clues regarding the direct role of complement system in the pathogenesis of ACPA-negative patients.

Recently, anti-CarP antibodies have been described in 16% ACPA-negative RA patients (196) and up to 46% patients with RA in various clinical studies. This led to the hypothesis that anti-CarP are closely related ACPA. ACPA recognize targets that are the result of the enzymatic process whereby arginine is converted into citrulline, while anti-CarP antibodies are the result of a chemical process in which lysine have been converted into homocitrulline (196). A few studies have shown that, similar to ACPA, anti-CarP antibodies are found before the onset of clinical symptoms of arthritis (119, 196). Anti-CarP antibodies have been found even in animal models of arthritis prior to the onset of disease, and its relevance will be discussed later (197). Furthermore, the significant association with radiological progression of anti-CarP IgG in ACPA-negative RA patients strongly suggested that anti-CarP antibody can also be used as a biological marker to diagnose a high risk RA population (196). Anti-CarP antibodies recognize many carbamylated antigens including human serum albumin, fibrinogen, and alpha-1 antitrypsin (196, 198–201). The comparative importance of anti-CarP vs. ACPA in the initiation of RA is unknown.

## Mouse Models of Human Rheumatoid Arthritis

Mouse models are commonly used to study human autoimmune diseases, including RA. Although mice do not develop arthritis naturally, arthritis that shares phenotypic, biochemical, physiological, and immunological properties similar to human RA can be induced in mice. Human RA often develops rapidly as an inflammation in one or more joints which is then followed by the development of the pannus. This acute inflammatory response to some extent has been replicated in some mouse models of inflammatory arthritis such as pristane-induced arthritis (202), zymosan-induced arthritis (203), proteoglycan-induced arthritis (204), streptococcal cell wall arthritis (205), the SKG mouse model of arthritis (206), and methylated bovine serum-induced arthritis (207). Several models are more adaptive immune-mediated or related, and include collagen-induced arthritis (CIA) (208), collagen antibody-induced arthritis (CAIA) (209), and the KBxN serum transfer model of arthritis (KBxN STA) (210, 211). Others are induced entirely by cytokines and include TNF-α transgenic mice (212) and IL-1Ra knockout mice (213). While some of these models are quite similar, in aggregate they possess different clinical, pathological, and mechanistic features that each representing a subset of the different aspects of human RA. With that caveat, here we will discuss the initiation and evolution of disease in three mouse models of human RA which are dependent on the complement system.

### Collagen-Induced Arthritis and Complement Activation

Approximately 50 years ago, CIA was first reported in rats following an intradermal injection of CII emulsified in Freund adjuvant (214), and later on in many susceptible strains of mice (208) as well as in non-human primates (215, 216). At present, CIA has become one gold standard mouse model of human RA and is used in many laboratories to examine the effect of therapeutics for treatment of RA. Immunization with bovine type II collagen/Freund’s complete adjuvant (CFA) or with chicken CII/CFA results in a severe polyarthritis disease after 3 weeks. Often a second injection is given on day 21, in some cases consisting of a second dose of CII/CFA and in some cases simply CII. The re-exposure to CII antigen continues to activate T and B cells and, in mice with the appropriate H2 alleles, creates an autoimmune disease attacking self-CII. In joints, CIA like human RA is characterized by the presence of activated synovial fibroblast like cells, pannus formation (multi-layered synovium), periosteal bone formation, cartilage surface damage, fibrin deposition, infiltration of macrophages and neutrophils, and finally ankylosis of one or more joints (208, 214). Similar to the anti-CII antibodies generated due to the presence of CII autoantigens in mice, similar antibodies to native or citrullinated CII are also present in human RA and appear to have pathophysiological significance (137, 140, 217).

In CIA, recombinant TNF-α induced an increase in anti-CII antibody levels indicating TNF-α contributes to disease development by both initiation of inflammation and production of autoantibodies (218). Anti-CII autoantibodies are generated in CIA mouse model arthritis and accumulated before the initiation of clinical signs of disease after the booster injection (219). This is somewhat similar situation in human RA where ACPA are present in the early evolution of RA before clinical signs of the disease, thereby suggesting that anti-CII or ACPA first accumulates to initiate the arthritis. In this regard, it has been observed that ACPA levels show an increase 3–5 years before the onset of clinical disease and then stabilize at a high levels (220). Perhaps, a certain threshold level of autoantibodies must be reached in a preclinical stage both in CIA and in human RA to develop disease. Additionally, in RA anti-CII autoantibodies were significantly associated with increased radiographic damage at the time of diagnosis (221).

It is interesting that not all strains of mice are equally susceptible to the CIA. Mice with the *H-2q* allele (MHC class II molecule) are highly susceptible to CIA, for example. It is thought that a particular immunodominant CII peptide region binds to this particular MHC allele with high affinity leading to a powerful anti-CII response (222–225). It is possible that a similar immunodominant CII peptide region binds to human RA associated allele HLA-DR (DR1*0401) (222, 226). The efficacy of abatacept (Orencia^®^), a fusion recombinant protein consisting of extracellular domain of T lymphocyte-associated antigen 4 linked to modified Fc of human IgG1, in human RA, clearly implicates T cell activity as important for disease progression, which is mirrored by the requirement for T cell help in CIA. Abatacept selectively inhibits T cell activation by two mechanisms, i.e., by blocking the specific interaction of CD80/CD86 receptors to CD28 and also by binding to CD80 and CD86 receptors on the antigen-presenting cells, thereby inhibiting B cell immune response. Immunization of mice with CII results in activation of CII-specific B cells followed by generation of IgG2a as a part of the humoral response (227, 228). The induction phase of CIA through activation of the CP leads to the activation of the adaptive immune response and the generation of anti-CII antibodies. These anti-CII antibodies then bind to cartilage, thereby leading to the effector phase *via* ICs formation and the activation of complement on the cartilage surface. So CIA pathology like human RA is dependent on both humoral and cell-mediated immunity (224, 229).

A vital role for complement in CIA was first suggested by studies in rats, in which injection of cobra venom factor (CVF), inducing marked activation followed by depletion of complement components, led to a delay in the onset of arthritis until serum C3 levels returned to normal (230). Both IgG and C3 are deposited on the cartilage surface in CIA (231), and C3 depletion (a.k.a. de-complementation) of recipient rats with CVF also prevented passive transfer of CIA with anti-collagen Ig (232). Pretreatment of rats with soluble complement receptor type 1 (sCR1), an inhibitor of the classical and AP C3 convertases, led to a delay in the development and progression of CIA (233). Whereas CVF could not alter the course of established CIA, sCR1 injections attenuated inflammation during active disease. Soluble CR1 binds to both C3b and C4b, leading to inhibition of C3 and C5 convertases and decreased activation of both C3 and C5. Furthermore, in CII-immunized mice, gene therapy with sCR1 delayed the development of CIA and decreased its severity (234). In addition, those mice expressing sCR1 exhibited decreased levels of anti-CII as well as markedly reduced lymph node and splenocyte proliferative responses to CII *in vitro*. Recent studies have shown that human TT32 (CR2-CR1), a potent CP and AP inhibitor, compared with human sCR1-10 attenuated CDA in mice with CIA (235). Normally, CR1 is expressed on many cells but a soluble form of CR1 is also present in human plasma therefore synthesized lacking the transmembrane and cytoplasmic domains (236). CR1 consists of four long homologous repeats (A–D), each containing seven SCR repeats (237). The first 10 SCR domains (1–10) of CR1 contain all important modalities required for pan-complement inhibition, acting as cofactors for irreversible proteolytic cleavage of C3b or C4b as well as decay accelerators for AP and CP convertases.

At that point, the relative importance of the CP or LP or AP of the complement system was not clear. One study clearly showed that complement activation by both the CP and the AP plays a deleterious role in CIA (224). Here, it was shown that *C3^−/−^* and *FB^−/−^* mice were highly resistant to CIA and demonstrated decreased CII-specific IgG Ab response. Repeated injection of CII for 3 weeks in *C3^−/−^* mice eventually resulted in the development of a low level of arthritis. Thus, C3 and FB deficiency ameliorate CIA, but do not fully protect against the development (224). Mouse complement receptor-related 1 gene/protein y (Crry), a C3 convertase inhibitor, plays a somewhat similar complement regulatory role as CR1. Transgenic mice overexpressing soluble Crry were generated and used for various complement related studies (238). Nonetheless, the AP activity in Crry-Tg mice was not inhibited as originally expected (238).

There was a suppression of CIA in Crry-Tg mice due to enhanced synthesis of Crry locally in the joint with decreased production of pro-inflammatory cytokines (239). The mice transgenic for Crry exhibited more inhibition of CIA than was recently observed in mice treated with a recombinant Crry-Ig fusion protein (219). It was concluded from these studies that the effects of Crry in CIA may be due both to inhibition of B cell function as well as to local blockade of production of pro-inflammatory cytokines.

More effective suppression of the complement system in disease may result from enhanced levels of complement regulatory proteins locally (knee joints) in tissues. Endogenous expression of complement regulatory proteins appears to be important in resistance to inflammatory disease as blockade of both Crry and CD59 led to more severe CIA in rats (240). These studies show that inhibition of an up-stream complement C3 or its C3 convertase can demonstrate profound effects on the initiation of CIA.

To show the role of downstream complement components such as C5 or C5a-C5aR axis in CIA, administration of anti-C5 inhibitory (BB5.1) antibody was used and was found to both prevent the initiation and decrease the severity of arthritis (219, 241). This inhibitory anti-C5 antibody prevented the cleavage of C5 into C5a and C5b, thereby blocking the terminal pathway. Furthermore, mice lacking C5 were partially resistant to CIA (242). However, in other studies, C5-deficient mice were not resistant to the CIA (243–245). So which component of the complement system C3 or C5 is important for the development of arthritis? Of the several components of complement, current evidence still points to the component C5-generated C5a as the strongest inducer of inflammation (246).

The greater inhibitory effects on CIA of an inhibitory anti-C5 antibody in comparison with Crry-Ig may be attributable to decreased levels of IL-1β and TNF α mRNA in the joints (219). To support ongoing clinical development and clinical trials, an inhibitory anti-C5aR monoclonal antibody not only completely inhibited the disease progression including reduced cartilage and bone destruction but also reduced TNF-α, IL-6, and IL-17A (247). Attempts have been made to design a recombinant vaccine to prevent CIA and also other mouse models of RA by inducing C5a-specific neutralizing antibodies without effecting C5/C5b (248). Injection of anti-rat CD59 induced spontaneous complement-dependent arthritis (240) and mice lacking CD59 are susceptible to antigen-induced arthritis (249). Thus, CIA is a valuable model of human RA to examine therapeutic intervention to block the upstream and downstream pathological by-products of the complement activation.

### Collagen Antibody-Induced Arthritis and Complement Activation

Collagen antibody-induced arthritis can be induced in mice by injecting a mixture of five monoclonal antibodies known as ArthritoMab™ or Arthrogen-CIA^®^ to different epitopes of the CII (250, 251). These antibodies binds to CII epitopes C11b, J1, D3, and U1 and spread across the entire CII region such as CB8, CB10, and CB11 fragments for better immune complex on the surface of cartilage to initiate arthritis.^1^,^2^ To induce CAIA in certain strains of mice and to get a 90–100% penetrance rate, the immunization with LPS following a mixture of four or five ArhritoMabs is essential (156). CAIA, like CIA, does not require the involvement of T and B cells for the priming phase and thus represents only the effector phase (252). There is evolving consensus that ACPAs predict the development of human RA (197, 253–255). It has been shown that arthritis can be introduced in mice by injecting a panel of mouse ACPAs (ACC1, ACC3, and ACC4) directed against citrullinated CII epitope (253, 256, 257). In a subset of human patients, ACPAs appears many years before the onset of disease. Furthermore, ACPAs from RA patients have been shown to activate complement *via* both the CP and the AP (179), but serum from RA patients failed to induce arthritis in mice. A somewhat similar experiment also failed to induce arthritis in DBA mice by transferring mouse mAbs against citrullinated fibrinogen (258). Nonetheless, these anti-citrullinated fibrinogen mAbs enhanced the suboptimal disease already established by the development of citrullinated antigens in the joint that are induced by the mixture of anti-CII (258). Thus, mouse models of RA clearly shows the importance of B cell generating anti-CII Abs or ACPAs which trigger the effector phase by activating the complement system. There is no mouse model of RA yet developed showing that anti-CarP autoantibodies such as anti-CII Abs can induce arthritis in mice through complement dependence. Nonetheless, similar to ACPA, their presence has been shown in mice and rhesus monkeys with arthritis (197, 200). Although the presence of ACPA in mice with arthritis is controversial, nonetheless the effector functions of anti-CII antibodies in mouse models have provided a clear picture of the pathophysiological processes or events likely to be involved in the initiation of human RA. One study has shown, using a rabbit arthritis model, that first anti-CarP antibodies might be generated from homocitrulline followed by ACPA (259). This observation alone related to the presence of anti-CarP before ACPA can have a huge impact to understanding the initiation of RA in ACPA-positive and ACPA-negative subset of patients.

It has been debated which pathway of the complement system is relevant in RA and how this pathway gets activated in human RA. CAIA using complement component gene-deficient mice has proven very useful to answer many of these questions. Some previous studies have shown that AP of the complement system is the main contributor because there were correlations between Bb and ICs levels (260, 261). The AP can be activated by IgA (261, 262) consistent with the current views regarding the mucosal (gut or lung) origin of the RA. Studies in CAIA mice have shown that the AP of complement is necessary and sufficient for the development of arthritis (263). In this study, C57BL/6 mice genetically deficient in either the AP protein FB (*FB*^−/−^) or in the CP component C4 (*C4*^−/−^) were used. CDA was markedly decreased in *FB*^−/−^ compared with wild-type (WT) mice. Conversely, disease activity scores were not different between *C4*^−/−^ and WT mice. Analyses of joints showed that C3 deposition, inflammation, pannus, cartilage, and bone damage scores were all significantly less in *FB*^−/−^ as compared with WT mice. There were significant decreases in mRNA levels of C3, C4, CR2, CR3, C3aR, and C5aR in the knees of *FB*^−/−^ as compared with *C4*^−/−^ and WT mice with arthritis; mRNA levels for complement regulatory proteins did not differ between the three strains. The authors concluded that the AP is absolutely required for the induction of arthritis following injection of anti-CII Abs (263). In a subsequent study, it was shown that arthritis was not altered in *C1q^−/−^* or *MBL A/C^−/−^* or in *C1q^−/−^/MBL A/C^−/−^* (no CP no LP) mice. These *in vivo* CAIA results proved the ability of the AP to carry out pathologic complement activation in the combined absence of intact CP and LP (71). In this study, C3 activation results confirmed the ability of the AP to mediate IC-induced C3 activation using sera from *C4^−/−^* or *C1q^−/−^/MBL A/C^−/−^* or both *C1q^−/−^/MBL A/C^−/−^* mice (71).

From these studies, it was concluded that the AP amplification loop, with its ability to greatly enhance C3 activation, is necessary to mediate inflammatory arthritis induced by adherent ICs. Then, it was questioned whether CP or LP alone mediate CAIA. Later on, it was reported that *FD^−/−^* (CP and LP), *C1q^−/−^/FD^−/−^* (no CP no AP), and *MBL A/C^−/−^/FD^−/−^* (no LP no AP) mice all these gene-deficient mice failed to develop to CAIA (264). One thing was common among these gene-deficient mice that there was lack of the AP of complement system. But whether AP is sufficient to initiate and sustain RA in humans is unknown. The AP alone on adherent anti-CII antibodies was capable of generating C5a to a level equal to that observed with WT sera. However, the CP alone, in the absence of the AP, generated 71% less C5a than was observed with WT sera; the LP alone generated minimal C5a (264). Huge activation of C5a using sera from *C1q^−/−^/MBL A/C^−/−^* (only AP) equivalent to sera from WT mice and their huge susceptibility to CAIA further suggested that enzymes/and or proteases independent from the MBL might be activating or regulating the AP (264). To this end, it was also confirmed by using *MBL A/C^−/−^/FCN A^−/−^*, and FCN A*^−/−^* mice that these ligands plays no role in CAIA (38). Mice lacking FCN B were partially protected while mice lacking Collectin 11 were susceptible to CAIA (10). FCN B is generated by the macrophages and macrophages infiltrate in the joints in mice with CAIA. These data solidify the role of LP as does the FCN B and MASP-1/3 in regulating the AP of the complement system.

While we have demonstrated that the AP plays a critical role in CAIA, we do not yet understand how it is activated in any molecular detail. Given our data, we suspect that factors independently from the LP ligands (MBL, FCN, and Collectins) such as such as MASPs might be activating the AP in CAIA.

Almost a decade ago, a landmark discovery was made regarding a LP enzyme, MASP-1/3 that entirely shifted the paradigm to the in-depth understanding of the interaction between the LP and AP of complement (265–267). It was shown by using *in vitro* studies that MASP-1/3 can cleave proFD (inactive) into FD (active). In this fashion, it seems that a MASP involved with the recognition of pathogens *via* the LP is also a critical activator of FD, a major component of the AP (265). Consistent with this observation, mice lacking the *MASP-1/3* gene have no LP and also have a defective AP (265, 266). Relating this to CAIA, we found that both *FD^−/−^* and *MASP1/3^−/−^* mice were resistant to CAIA (268) and there was no change in the status of proFD in *MASP-1/3^−/−^* mice before or after the induction of CAIA (38), confirming the *in vivo* role of MASP-1/3 in the cleavage of proFD into FD. *In vitro*, adherent anti-CII antibodies failed to fully restore C3 activation using AP-defective sera from *MASP-1/3^−/−^* or *FD^−/−^* mice (268) consistent with the *in vivo* CAIA resistance. It was further shown, using *ex vivo* cartilage microparticles (CMP), that MASP-1/3 proteases can cleave proFD in the knee joint microenvironment (269). Here, cultured differentiated 3T3 adipocytes were used as a surrogate for synovial adipose tissue. They produce proFD but not mature FD. On the other hand, fibroblast-like synoviocytes (FLS) derived from CIA synovium, were the main source of MASP-1/3 and were expected to process proFD to mature FD. Using CMP coated with anti-CII mAb and serum from *MASP-1/3^−/−^* mice as a source of FB, proFD in 3T3 supernatants was cleaved into mature FD by MASP-1/3 in FLS supernatants. The mature FD was eluted from the CMP and was not present in the supernatants from the incubation with CMP, indicating that cleavage of proFD into mature FD by MASP-1/3 occurred on the CMP. These results demonstrated that pathogenic activation of the AP may occur in the joint through IC adherent to cartilage along with the local production of necessary AP proteins by adipocytes and FLS (269). To provide another proof-of-concept experiment, *in vivo* reconstitution of MASP-1 or MASP-3, by liver derived from *FD^−/−^* mice, transplanted under the kidney capsule of *MASP-1/3^−/−^* mice and restored the cleavage of proFD into FD in the circulation of *MASP-1/3^−/−^* mice (270). Consistent with this, we found that sera from *MASP-1/3^−/−^* mice, which have defective AP, only after transplantation restored the full AP activity (270). These data confirmed that MASP-1/3 proteases of the LP are essential for the activation of the AP in mice. A new concept evolved from these studies that liver (generating MASPs) and adipose tissue (generating proFD) might acts in concert to activate the AP, thereby playing a vital role in the development of CAIA in mice (38, 43, 264, 270).

There are a number of ligands which can activate the LP. Presumably, the LP contribution to CAIA involves a subset of ligand interactions. Known candidates include MBL A, MBL C, FCN A, FCN B, and collectin 11. To address this, we examined CAIA in *FCN A^−/−^, FCN B^−/−^*, and *CL-11^−/−^* mice as mentioned earlier (10). These studies showed the important role of FCN B ligand of the LP in directly activating MASP-1 or MASP-3 to activate the AP. By contrast, we also observed partial protection in *MASP-2/sMAp^−/−^* mice. This was likely due to the involvement of the C4/C2 bypass pathway in CAIA (10). Given that MASP-1 and MASP-3 are splice variants derived from a single MASP-1/3 gene, it has been difficult to separate the two functionally. Nonetheless, our most recent data suggest that MASP-3, compared with MASP-1 or MASP-2, is the main driver of the AP and thus CAIA (271). In these studies, MASP-3 siRNA inhibited CAIA compared with MASP-1 or MASP-2 siRNAs (271). All of the above, *in vivo* CAIA studies, show that MASP-3 proteases of the LP regulate the AP, a finding that has also been confirmed by using *in vitro* studies by various research groups (44, 45, 265, 268, 272). There is a possibility that this amplification driven phenomena is surface-specific or disease specific as MASP-1/3 deficiency did affect kidney pathology in *MASP-1/3^−/−^/FH^−/−^* mice (273) whether this kidney pathology is different from *FD^−/−^* mice is unknown. It has been reported that mechanism of AP activation depends on the activator surface (47). Here, it was shown that MASP-1 inhibition prevent AP activation as well as prevent already initiated AP activity on the LPS surface but not for zymosan-induced AP activation (47). Overall, it appears that AP is not one holistic linear pathway as previously thought but it is an interwoven network of multiple pathways regulated by the LP enzymes.

Using CAIA, the effector roles of C3aR and the C3a–C3aR axis, C5aR, and the C5a–C5aR axis, and MAC deposition have also been dissected. Mice lacking C5aR were more resistant to CAIA than C3aR- or MAC-deficient mice, confirming the pivotal role C5–C5aR axis (274). These results are consistent with the concept of the predominant role of C5 over the role of C3 in the pathogenesis of CAIA and that the C5–C5aR axis is essential for CAIA, although C3aR and the MAC also played important roles. Consistent with this conclusion, *C3^−/−^* mice were partially protected from CAIA (71) while *C5^−/−^* failed to develop CAIA (242).

By contrast, why the inhibitory anti-human C5 antibody (Eculizimab, Soliris^®^) was not effective against RA in clinical trials is not known. There is a possibility that C5 is generated in high quantities in RA, so even high doses of antibody do not prevent C5a generation in the joints. Alternatively, it may be the case that complement plays a more important role early in disease and that eventually RA evolves to a state where complement is only one of several drivers that can each compensate for the other. In this scenario, it might be the case that only certain RA patients would be effectively treated by Eculizimab. While the liver is the major source of C5, neutrophils, macrophages, and T cells are all known to be sources of C5. Blocking of C5aR in human neutrophils using the small molecule inhibitor; PMX-53, resulted in a dose-dependent block of C5a-mediated activation but why it was unsuccessful and very disappointing in RA clinical trial is unknown (275). Possibly, the drug was cleared rapidly and never reached the joints. No doubt that preclinical studies support targeting C5aR in RA because C5a and C5aR are elevated in the joints of RA and psoriatic arthritis patients and their blockade attenuate leukocyte migration to the synovial fluid (276). Almost complete inhibition of CIA was reported using anti-mC5aR inhibitory antibody (247). Based on these preclinical studies, a fully human antibody that blocks the binding of C5a to C5aR was developed and tested in RA patients by Novo Nordisk, a pharmaceutical company. This company has conducted two Phase I clinical trials in Europe with anti-C5aR in patients with RA, where a good drug safety profile was demonstrated.^3^ Further results regarding the success or failure related to the phase II clinical trials by Novo Nordisk using anti-C5aR therapeutic antibody in RA patients are unknown at this points. Therefore, it is too early to make any conclusions regarding the therapeutic use of anti-C5aR antibody in the clinical settings. Furthermore, mice treated with GalNAc C5siRNAs targeting liver C5 are resistant to arthritis (277). Recently, with a new approach, CAIA mice injected with anti-C5aR antibody conjugated with C5siRNA inhibited arthritis in mice identical to the *C5aR^−/−^* mice with an inhibition of more than 80% of the disease (278). These results in CAIA are promising, showing selective and simultaneous inhibition of both C5aR activity and C5 mRNA production within the C5a–C5aR axis can dampen inflammatory response and attenuate arthritis in mice. Intriguingly, it has been shown in an experimental mouse model of autoimmune hemolytic anemia that C5aR activation does not necessarily involve C5 and C5a (279). This striking observation suggests that we must also consider the coordinate modulation of the FcγR system when interpreting the role of C5aR in RA.

Factor H is known to regulate the AP and due to the concurrent absence of C3 through uncontrolled complement activation in the fluid phase, *FH^−/−^* mice are resistant to CAIA (156). There is a variant of human FH gene, i.e., factor H-like-1 (FHL-1). There are five different forms of FHR proteins in humans (FHR-1 to FHR-5) (a.k.a. complement factor H-related proteins). Of these, FHL-1 and FHR are believed to counteract the effects of FH. At present, little is known of the effect of FHL-1 or FHR on CAIA due to the lack of experimental models. FH and FHL-1 have been shown to be expressed and secreted by synovial fibroblasts and were present in synovial fluid derived from patients suffering from rheumatoid or reactive arthritis (280). Endogenous FH is capable of inhibiting activation of the AP of complement on cartilage and synovium in joints *in vivo* exposed to a submaximal level of anti-CII mAb (156). This conclusion was derived from experiments in CAIA, with mice treated with rFH19-20 to prevent engagement of full-length endogenous FH. This takes advantage of the observation that domains 19 and 20 of rFH bind to cartilage. By treating with rFH19-20, the interaction of FH with cartilage is inhibited. To evaluate the *in vivo* importance, it was found that competitive blockade by murine rFH19-20 of the binding of endogenous fluid phase FH to either cartilage or an injured FLS surface significantly increased CAIA in WT mice. Further support for the conclusion that FH plays a key role in regulating AP-induced complement deposition on cartilage and cell surfaces in the joint is derived from studies with *FH^±^* heterozygous-deficient mice. These mice exhibit lower circulating levels of FH but are not more susceptible to CAIA unless they are treated with rFH19-20 to disrupt tissue binding of endogenous FH. Recombinant fH19-20 impairs only surface control of the AP by FH and does not influence the systemic activation of the complement system as indicated by unchanged serum levels of C5a. Thus, FH controls AP activation on cartilage and injured FLS *in vivo* in a manner dependent on the FH SCR19-20 domain, indicating that the AP can be regulated on these joint surfaces. Mice lacking FH do not develop arthritis due to the lacking of C3 present in the circulation (156).

In contrast to FH, there are no studies showing the direct role of mouse FHR proteins which shows some sequence homologies to FH in the pathogenesis of inflammatory arthritis in mice. In mice, various transcripts of FHR proteins have been reported such as FHR-A, FHR-B, and FHR-C (278, 281, 282). One study has shown that recombinant mouse FHR-B bound to human C3b and was able to compete with human FH for C3b binding. FHR-B supported the assembly of AP convertase *via* its interaction with C3b. The authors concluded that mouse FHR-B similar to human FHR-1 and FHR-5 promoted complement activation *via* interaction with C3b and *via* competition with mouse FH (283). Similarly, it has been shown that mouse FHR-A and mouse FHR-B proteins antagonize the protective function of FH using sheep erythrocyte hemolytic assays and in two cell lines, kidney proximal tubular cell line and a human retinal pigment epithelial cell line (ARPE-19) (284). Lack of mouse FHR-C has been linked to an autoimmune disease (278). Still none of these above FHR studies have shown the direct role mouse FHR-A, FHR-B and FHR-C in mice with arthritis for *FH^−/−^* are resistance to CAIA and depletion of C3 in these mice occurs in *FH^−/−^* mice even in the presence of all mouse FHR proteins when there is no absolute competition.

These findings related to the role of AP in CAIA are very likely to be relevant to the initiation and perpetuation of arthritis in humans (103). Recent preclinical studies have shown that human TT32 (CR2-CR1), a potent CP and AP inhibitor, compared with control human sCR1-10 also significantly attenuated CDA in mice with CAIA (235). In man, circulating autoantibodies, including anti-CII Abs, are present for several years prior to the onset of clinically apparent arthritis (221). Substantial evidence suggests that in RA joint-based inflammation is initiated through Ag/Ab complexes that are present on the cartilage surface (285). The observation that only injured FLS, but not normal FLS expressing complement regulatory proteins, could exhibit C3 binding suggests that cartilage damage may precede injury to the synovium. Initial complement activation by solid phase immune complexes in the cartilage may lead to secondary damage to the FLS and thus to subsequent development of synovitis. Therefore, potent CP and AP inhibitors might be helpful clinically to attenuate cartilage damage seen in human RA. This strategy of using complement inhibitors can be very useful during the early development of RA because once ACPA antibodies are present in subjects without clinical signs of joints damage then there are 50% chances of developing RA with 3-year period. Such clinical trials “Strategy for the Prevention of Onset of Clinically-Apparent RA” or a.k.a. StopRA^4^ are already in progress.

### K/BxN Serum Transfer Mouse Model of Arthritis and Complement Activation

About 20 years ago, an additional mouse model of RA, i.e., K/BxN serum transfer arthritis (STA) was discovered (210). It is also being used extensively to examine the role of effector pathways of the autoantibodies. This RA mouse model is different from the CIA and CAIA models as disease is driven by activation of T cells that recognize a self-peptide (i.e., glucose-6-phosphate isomerase, G6PI) (286). These T cells then help B cells to generate IgG antibodies against G6PI which induces arthritis. Furthermore, either purified IgGs or serum alone from K/BxN arthritic mice, when injected into naïve mice, is capable of inducing severe arthritis (287). So G6PI autoantibodies target the G6PI antigen in the joints thereby inducing arthritis by binding to cartilage. In this model, a pooled serum from several arthritic K/BxN mice is transferred into naïve mice to induce arthritis. The isotype of G6PI autoantibodies is IgG1 which does not activate complement as compared to the anti-CII antibodies used for CAIA (288). Whether G6PI antibodies present in RA patients have any practical diagnostic value is unknown. One study has shown the presence of anti-G6PI antibodies in sera but there were no marked differences in the levels of anti-G6PI antibodies among RA, non-RA patients, and healthy controls. Also, there was no significant difference G6PI antibody levels between the active phase and the inactive phase in RA patients (289). It is also controversial as to whether synovial fibroblasts from RA patients can secrete G6PI. One study has been published showing the presence of a distinct population of cells at the surface of the synovial lining of inflamed RA joints that has a high concentration of G6PI (290). This cell population could be T cells present in the RA synovium (291). Interestingly, serum G6PI concentration, C1q/G6PI-CIC, and G6PI mRNA levels within peripheral blood mononuclear cells were significantly higher in active RA than that in non-active RA (292). This is controversial for it has been shown that G6PI is not a specific autoantigen in RA and only few autoimmune sera contains G6PI (293, 294).

Using the K/BxN STA mouse model, the severity of inflammation has been correlated with the expression of PAD2 and PAD4 in the close proximity of citrullinated fibrinogen (295). Two isotypes of PAD2 and PAD4 have been shown to be highly expressed in the synovium of RA patients (295) and infiltrating cells neutrophils, macrophages, and mast cells are the major source these enzymes indicating local citrullination in the joints can take place. Moreover, anti-PAD4 autoantibodies are present in a subset of RA patients (296). Perhaps these autoantibodies are generated to inhibit the excessive conversion of arginine to citrulline as a defensive mechanism. Interestingly, although PAD4 is required for citrullination, PAD4-deficient mice were not protected from arthritis in the K/BxN STA model (295). Once again, human RA clinical studies reflect a different picture of the GPI autoantibodies and also the role PAD4 than the mouse models of K/BxN STA.

Despite the lack of activation of complement by anti-G6PI antibodies, it is fascinating to note that complement activation is still required for K/BxN mice to progress to RA. Studies have examined mice lacking complement components, C3 or FB or C5 in the context of the K/BxN model and have established that these genes are required for disease development (287, 297, 298), thereby showing that the AP of complement is required. Mice lacking C1q, C4, CR1, and CR2 remained susceptible to disease development in the context of K/BxN STA (287, 297, 299). These studies have shown that CP is not required for disease progression in K/BxN STA. Properdin deficiency rescued mice from complement-mediated injury and ameliorated disease in K/BxN STA and Ab neutralization of properdin in WT mice similarly protected mice from arthritis (300). Mice lacking MAC also were not protected using K/BxN STA (287) showing that MAC is not a significant mediator of disease in this model. By contrast, C6 deficiency has been shown to partially protect mice from CAIA (274) indicating MAC, i.e., the terminal pathways of the complement can play important role. Overall, K/BxN STA have provided very important information regarding the role complement in RA and illustrates the complex nature of human RA. Furthermore, most of the data in K/BxN STA are consistent with CAIA regarding the role of AP in the initiation of arthritis.

## Mouse Models of Human Rheumatoid Arthritis and Complement in the Present and Future

Human RA is a complex disease. This becomes readily apparent when one considers that some patients respond well to TNF-α blockade while others do not and instead respond to T or B cell inhibition. In this regard then, it is useful to have multiple mouse models, each of which uses a different driver to ultimately produce synovitis. Given that all three models require a functional complement system, it suggests that the actions of complement are fundamental components of disease progression.

We might divide the process of disease progression in RA into two general subprocesses: initiation and the effector phase. During initiation, autoantibodies find their way to the cartilage and synovial space. These may be generated in response to a pathogen such as *P. gingivalis* or as a response to altered self (i.e., citrullination). ACPA or anti-CarP antibodies in human patients are detected years before disease becomes apparent. In CIA, bovine CII is introduced causing the production initially of anti-bovine CII and then of anti-self-CII. In CAIA, anti-self-CII antibodies are directly introduced. We believe that these autoantibodies serve to initiate the complement cascade either through the CP or the LP. This early activation of complement then initiates an inflammatory response *via* the production of C3a and C5a. As the autoantibodies are located in the joint space, the response is synovitis. The epitope to which the antibody is directed appears to be mutable. Thus, CIA mouse model replicate somewhat an identical chain of early events mostly in RA patients in the initial phases of arthritis. While, ACPA is present in many RA patients, there exist a population of ACPA-negative RA patients which presumably have initiated disease *via* a different mechanism. Although this finding challenges a universal pathogenic model for a key role of autoantibodies in all types of RA, but our preliminary data from CAIA show that even a tiny amount of anti-CII autoantibodies can still bind to the cartilage surface when these autoantibodies are completely absent in the circulation. Therefore, one cannot rule out the presence of a very low levels, i.e., below threshold levels of ACPA or anti-CarP or anti-CII autoantibodies in other body secretions such as nasal secretions or sputum or gingival crevicular fluid or saliva, when autoantibodies are completely absent in the circulation.

Once other immune cells have infiltrated the joint, synoviocytes have proliferated, and pannus has formed, RA has entered into the effector phase. Here, pannus secretes matrix metalloproteinases which act to destroy bone and cartilage while also secreting a complex mixture of cytokines, prostaglandins, and complement components to maintain the inflammatory state. Complement can play a role here as well, although this may be less prominent. Such a later stage role can be seen in the K/BxN STA model of RA. Anti-G6PD antibodies are of the IgG1 type which do not serve to activate complement. Thus here, RA is initiated by a different mechanism. However, as discussed above, components of the AP appear to be necessary for disease in this model. We suspect that in this model complement is required for the formation of pannus and thus acts in the effector phase of RA. Indeed, we find that components of the AP are essential for CAIA to progress as well.

Considering commonalities among the various mouse models of RA, it seems that the AP of complement is universally shared. Here FD cleaves and activates FB, which in turn is necessary for the formation of the C3 convertase on surfaces to amplify the complement response. MASP-3 generated by the liver has recently been identified as the protease critical for the cleavage of proFD and thus for the activation of the AP. In this regard, it we believe that MASP-3 may serve as an important clinical target for the treatment of human RA.

## Author Contributions

Both authors listed have made a substantial, direct, and intellectual contribution to the work and approved it for publication.

## Conflict of Interest Statement

The authors declare that the research was conducted in the absence of any commercial or financial relationships that could be construed as a potential conflict of interest.

## Footnotes

^1^www.mdbioproducts.com (Accessed: March 26, 2018).

^2^www.chondrex.com (Accessed: March 26, 2018).

^3^http://ClinicalTrials.gov Identifier: NCT02151409 (Accessed: March 26, 2018).

^4^http://ClinicalTrials.gov NCT02603146 (Accessed: March 26, 2018).

## Abbreviations

CP, classical pathway; LP, lectin pathway; MBL, mannose-binding lectin; AP, alternative pathway; CICs, circulating immune complexes; CRDs, carbohydrate recognition domains; FLS, fibroblast-like synoviocytes; CII, bovine type II collagen; PAD2 or PAD4, peptidyl arginine deiminase type 2 or 4; CDA, clinical disease activity; RF, rheumatoid factor; ACPA, anti-citrullinated protein antibodies; anti-CarP, anti-carbamylated protein; CIA, collagen-induced arthritis; PIA, pristane-induced arthritis; ZIA, zymosan-induced arthritis; PGIA, proteoglycan-induced arthritis; SCWA, streptococcal cell wall arthritis; CAIA, collagen antibody-induced arthritis; mBSA, methylated bovine serum-induced arthritis; KBxN STA, KBxN serum transfer model of arthritis; Crry, mouse complement receptor-related gene y; MASP-1, mannose-binding lectin-associated serine protease-1; MASP-2, mannose-binding lectin-associated serine protease-2; MASP-3, mannose-binding lectin-associated serine protease-3; MAp44, mannose-binding lectin-associated protein of 44 kDa a.k.a. MBL/ficolin/CL-11-associated protein-1 (MAP-1); FCNs, ficolins; FCN A, ficolin A; FCN B, ficolin B; CL-11, collectin liver 11; mAb, monoclonal antibody; MAC, membrane attack complex.
